# Effects of the Prolong Life With Nine Turn-Method Qigong on Fatigue, Insomnia, Anxiety, and Gastrointestinal Disorders in Patients With Chronic Fatigue Syndrome: Protocol for a Randomized Controlled Trial

**DOI:** 10.2196/53347

**Published:** 2024-02-26

**Authors:** Fangfang Xie, Yanli You, Yuanjia Gu, Jiatuo Xu, Fei Yao

**Affiliations:** 1 Shanghai Municipal Hospital of Traditional Chinese Medicine Shanghai University of Traditional Chinese Medicine 200071 Shanghai China; 2 Shanghai University of Traditional Chinese Medicine 201203 1200 Cailun Road, Pudong New District, Shanghai 201203, China Shanghai China; 3 ChangHai Hospital Naval Medical University 200071 Shanghai China

**Keywords:** chronic fatigue syndrome, prolong life with nine turn method Qigong, fMRI, gut microbiota, gastrointestinal, fatigue, insomnia, CFS, study protocol, Qigong, efficacy, safety, cognitive behavioral therapy, CBT, randomized trial

## Abstract

**Background:**

Chronic fatigue syndrome (CFS) is a debilitating multisystem disorder that can lead to various pathophysiological abnormalities and symptoms, including insomnia, gastrointestinal disorders, and anxiety. Due to the side effects of currently available drugs, there is a growing need for safe and effective nondrug therapies. The Prolong Life With Nine Turn (PLWNT) Qigong method is a system of mind-body exercise with restorative benefits that can alleviate the clinical symptoms of CFS and impart a significant inhibitory effect. Various studies have proven the treatment efficacy of PLWNT; however, the impact on insomnia, gastrointestinal disorders, and anxiety in patients with CFS has not yet been investigated.

**Objective:**

This study aims to evaluate the efficacy and safety of the PLWNT method in terms of its effects on fatigue, insomnia, anxiety, and gastrointestinal symptoms in patients with CFS.

**Methods:**

We will conduct a randomized, analyst-blinded, parallel-controlled trial with a 12-week intervention and 8-week follow-up. A total of 208 patients of age 20-60 years will be recruited. The patients will be randomly divided into a PLWNT Qigong exercise group (PLWNT Group) and a control group treated with cognitive behavioral therapy at a ratio of 1:1. Participants from the treatment groups will be taught by a highly qualified professor at the Shanghai University of Traditional Chinese Medicine once a week and will be supervised via web during the remaining 6 days at home, over 12 consecutive weeks. The primary outcome will be the Multidimensional Fatigue Inventory 20, while the secondary outcomes include the Pittsburgh Sleep Quality Index, Gastrointestinal Symptom Rating Scale, Hospital Anxiety and Depression Scale, functional magnetic resonance imaging, gut microbiota, and peripheral blood.

**Results:**

The study was approved by the ethics committee of Shanghai Municipal Hospital of Traditional Chinese Medicine in March 2022 (Ethics Approval Number 2022SHL-KY-05). Recruitment started in July 2022. The intervention is scheduled to be completed in December 2024, and data collection will be completed by the end of January 2025. Over the 3-year recruitment period, 208 participants will be recruited. Data management is still in progress; therefore, data analysis has yet to be performed.

**Conclusions:**

This randomized trial will evaluate the effectiveness of the PLWNT method in relieving fatigue, insomnia, anxiety, and gastrointestinal symptoms in patients with CFS. If proven effective, it will provide a promising alternative intervention for patients with CFS.

**Trial Registration:**

China Clinical Trials Registry ChiCTR2200061229; https://www.chictr.org.cn/showproj.html?proj=162803

**International Registered Report Identifier (IRRID):**

PRR1-10.2196/53347

## Introduction

Chronic fatigue syndrome (CFS) is a complex neuroimmune disease, usually characterized by severe fatigue, cognitive and autonomic dysfunction, and gastrointestinal discomfort, which seriously affect activities of daily living [1]. Mental sickness, sleep disorders, and gastrointestinal dysfunction are common symptoms reported by patients with CFS [2]. It is estimated that the average prevalence of adult CFS worldwide is approximately 0.65%, which rises to 0.89% if the most commonly used case definition is used [3]. Among patients with CFS, 82%-95% of people experience lifelong symptoms of mental and sleep disorders [4] and have a high need for health care [5]. In the general population in the United States and the United Kingdom, the prevalence of CFS ranges from 0.4% to 2.5% [6,7]. In 2015, the prevalence of CFS in the US population was approximately 836,000 to 2.5 million, among whom 42.2% and 33.3% of patients with CFS presented with anxiety and depression, respectively, according to data from the Institute of Medicine [8,9]. CFS has a greater negative impact on the patient’s physical and mental health.

In addition to neurological disorders, common symptoms reported by patients with CFS are characteristic of gastrointestinal diseases and are often accompanied by gastrointestinal symptoms such as nausea and vomiting [10,11]. Investigation into drug use in patients with CFS revealed that the use of antacids, H2 blockers, and proton pump inhibitors for the intestinal discomfort associated with CFS has increased significantly [12]. From a medical perspective, CFS is clinically regarded as part of a series of disease symptoms, including fibromyalgia and irritable bowel syndrome, and it may be related to the influence of the gastrointestinal digestive system or autoimmune symptoms [13]. Further evaluation of the role of intestinal flora in CFS neurological symptoms expression has suggested higher levels of D-lactic acid–producing enterococci and streptococci in the intestinal flora of patients with CFS compared with healthy controls [14]. The acute manifestations of D-lactic acidosis overlap with the psychiatric symptoms of CFS, prompting the use of drugs that target the commensal enteric microbiota as a possible treatment for the neurological symptoms of CFS [15]. Increasing evidence shows that CFS gastrointestinal symptoms may be related to various other symptoms, including, but not limited to, neurological symptoms. Thus, microbial imbalances should not be viewed in isolation [14]. Previous studies have indicated that nerve and gastrointestinal symptoms of CFS may be caused by food, such as dairy products and alcohol [16,17]. Neurological and gastrointestinal symptoms decrease during fasting, which is shown to be related to disruption of the brain-gut interaction [18]. It has also been reported that the traditional Chinese medicine Tianma, which is often used to treat headaches, not only relieves CFS but also regulates the intestinal flora [19]. This suggests that the brain-gut interaction plays a key role in CFS. The gut-brain interaction occurs through a variety of two-way pathways, including the central and enteric nervous systems or neuroimmune pathways and intestinal microbiota [20,21].

Both drug and nondrug therapies have been used for the prevention and treatment of CFS. Symptomatic treatment is often used in clinical treatment, including nonsteroidal anti-inflammatory drugs, exercise therapy, and cognitive behavioral therapy (CBT), which can relieve gastrointestinal and mental symptoms and improve the quality of life. The latest CFS treatment guidelines emphasize the change in the overall treatment attitude of CFS; for instance, drug treatment is now regarded as an adjuvant treatment of CFS nonpharmacological intervention [22]. Several researchers have proposed that graded exercise therapy and CBT might be effective treatments for CFS to improve fatigue and poor mental health, including depression, anxiety, and schizophrenia [23,24]. However, evidence of persistent and sustained substantial outcomes in patients with CFS is insufficient [25]. The clinical manual published by the International Association of Chronic Fatigue Syndrome recommends traditional Chinese medicine treatments, including acupuncture, massage, and Qigong. Qigong, as an intervention with proven safety and efficacy, has been recommended for the control of gastrointestinal diseases according to the clinical practice guidelines developed by the expert working group of the Sociedad Espanola de Patologia Digestive [26,27].

As one of the traditional Qigong (pronounced “chee gun”) for optimizing and restoring physical and mental energy, the prolong life with nine turn (PLWNT) method has a history of thousands of years, which was circulated by a centenarian named Kai Fang in the Qing Dynasty [28]. The PLWNT method, which can be practiced either statically (sitting, lying, and standing) or dynamically (moving), includes 8 kinds of massage manipulations on the abdomen and a kind of upper body shaking [29]. The abdominal massage techniques act on the movement of the pelvic and abdominal muscles, coordinated with diaphragmatic breathing. This may trigger the contraction of the intestinal and rectal muscles [30], which can train the function of the intestines [31] and impact the nervous system by reducing the excitability of the sympathetic nerve and enhancing the excitability of the parasympathetic nerve to reduce anxiety when rubbing the internal organs [32,33]. Long-term practice of PLWNT helps relieve the disease and regulate the functional activities of the meridians and viscera [34,35]. Moreover, exercisers can achieve “relaxation of mind and body” and “unity of nature and man,” thereby experiencing emotional stability and enhancing physical strength and health [36-38]. In our previous studies [39], it was confirmed that PLWNT is effective in reducing fatigue and improving the quality of life; however, whether the PLWNT has gastrointestinal, sleep, and emotional effects and the mechanism underlying these effects remains unclear.

In recent years, the brain-gut axis has been found to play an increasing role in the pathogenesis of CFS. The dysfunction of the central nervous system in processing gastrointestinal signals is considered to be a cause of CFS [40,41]. Moreover, previous studies have shown obvious abnormalities in the brain function activities and the species diversity of the gut microbiota in patients with CFS [42,43]. However, there has been no previous report on the use of PLWNT Qigong therapy to prove whether it can alleviate the clinical symptoms and regulate brain-gut interaction in patients with CFS. Therefore, we plan to use functional magnetic resonance imaging (fMRI) to analyze brain activity and connectivity data and collect stool samples to analyze the composition of the intestinal microbiota in patients with CFS.

This randomized controlled trial will be conducted based on the hypothesis that the application of PLWNT could alleviate fatigue, insomnia, anxiety, and gastrointestinal symptoms in patients with CFS. The study objectives are as follows: (1) to verify that PLWNT has a positive effect on fatigue, insomnia, anxiety, and gastrointestinal symptoms in patients with CFS compared with CBT, and (2) to fully explore the mechanism underlying the brain-gut regulation in CFS.

## Methods

### Study Design

**Figure 1:**
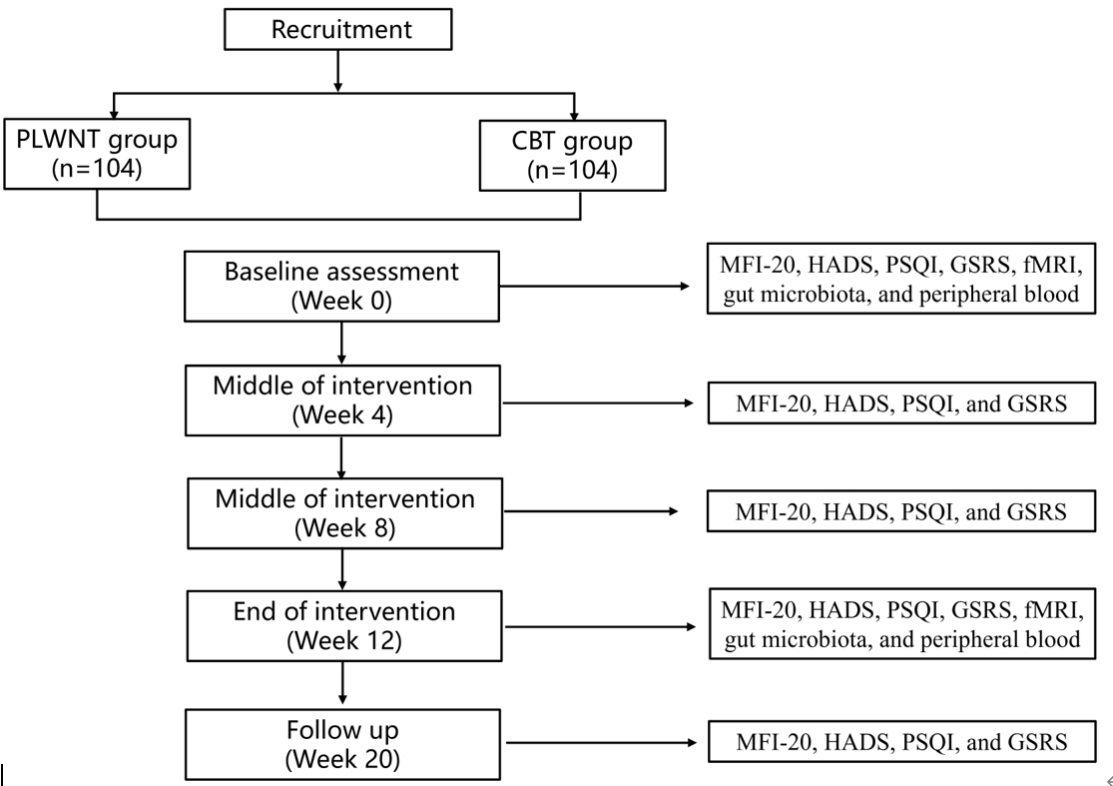
Flow diagram of the study design. CBT: cognitive behavioral therapy; fMRI: functional magnetic resonance imaging; GSRS: Gastrointestinal Symptom Rating Scale; HADS: Hospital Anxiety and Depression Scale; MFI-20: Multidimensional Fatigue Inventory 20; PLWNT: prolong life with nine turn; PSQI: Pittsburgh Sleep Quality Index.

A total of 208 patients with CFS who meet the requirements will be randomly assigned to the PLWNT group and CBT group. The PLWNT group will be treated with Qigong, and the control group will be treated with CBT (Figure 1). Patients will focus on offline practice once a week and via web practice at home 6 times a week for 12 weeks. The study protocol was developed in accordance with the SPIRIT (Standard Protocol Items: Recommendations for Intervention Trials) [44].

### Participant Recruitment and Informed Consent

A total of 208 participants will be recruited via WeChat (Tencent co, Ltd) or posters positioned at the Shanghai Municipal Hospital of Traditional Chinese Medicine from July 2022 to December 2024 in Shanghai, China. We will also include hospitalized patients with a preliminary diagnosis of CFS, according to the latest guidelines for the treatment of CFS revised in 2021 [45]. Before the commencement of the study, information on the study protocol will be provided to each participant. Participants will be briefed on the study process and informed of their responsibilities, and they will be informed that participation is entirely voluntary and that they may withdraw at any time. Once a participant has withdrawn, the collected data will not be deleted and will be used in the final analyses. Written informed consent will also be obtained from participants for this study, and the research assistant will be responsible for obtaining informed consent from all participants.

### Eligibility Criteria

#### Diagnostic Criteria

According to the latest NICE guidelines for 2021 [46], participants diagnosed with CFS must meet the criteria of debilitating fatigue worsened by activity, postexertional malaise, cognitive difficulties, and unresolved fatigue on awakening from sleep. All 4 symptoms must be present and have lasted for at least 3 months.

#### Inclusion Criteria

The study inclusion criteria are as follows: (1) age between 20 and 60 years; male or female; (2) severe chronic fatigue lasting at least 3 months, unexplained after clinical evaluation, not caused by work performed during the trial, and unable to be alleviated after rest; (3) meet the criteria of debilitating fatigue worsened by activity, postexertional malaise, cognitive difficulties, and unresolved fatigue upon awakening from sleep; (4) normal blood, urine, liver, and kidney functions; and (5) participants were not taking any other medication for 1 month prior to enrollment in this study and were not on any other treatment regimen.

#### Exclusion Criteria

The exclusion criteria are as follows: (1) no fatigue complaints or fewer than 4 symptoms; (2) severe cardiovascular, cerebrovascular, endocrine, motor, autoimmune, or infectious diseases; (3) pregnant or lactating women, drug addiction, heavy metal poisoning, or other similar conditions; (4) use of medications that may affect the judgment of the results; and (5) the participant requested withdrawal of informed consent.

### Randomization and Allocation Concealment

Patients with CFS who meet the criteria and sign the consent form will be randomized. A statistician who did not participate in the trial process will use computer software (Strategic Applications software, version 9.1.3; SAS Institute Inc) to create a random number table, which will be sent to a designated administrative staff member at the Test Management Center of Shanghai University of Traditional Chinese Medicine to ensure safety. They do not participate in the trial recruitment or treatment of participants. The administrative staff will store the patient’s identity information, treatment method, time, and location in an opaque envelope based on random numbers, before handing the sealed envelope to the research team, who will print and save it in its original form.

### Blinding

A double-blind study is difficult to implement in patients with CFS due to the difference between the 2 treatment methods. To reduce the influence of subjective factors of the patients and researchers on the study results, the Qigong and psychologists in charge of treatment will not participate in data collection, analysis, and result evaluation. Additionally, we will ensure that the evaluation and statistical analysis are performed by statistical analysts who are blinded to the grouping and intervention procedures. If the patient has an adverse reaction and requires suspension or termination of the trial, we will immediately stop the treatment and break the blind code.

### Sample Size Calculation

According to our published paper, the efficacy of the PLWNT group is assumed to be better than that of the CBT group. Referring to our previously published study [47] on the efficacy of CFS with respect to the Multidimensional Fatigue Inventory 20 (MFI-20) scale, the final difference between the 2 groups in terms of MFI-20 average scores was 1.634 and the SD was 3.238. The Bonferroni conservative comparison method was used, and the sample size of this trial was calculated using the following formula:

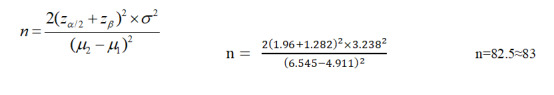



Considering the allowable 20% dropout rate, the sample size of each group was set at 104. Therefore, this randomized controlled trial needed to recruit 208 participants.

### Intervention

We will implement intervention measures in strict accordance with the CONSORT (Consolidated Standards of Reporting Trials) [48] and the STRICTA (Standards for Reporting Interventions in Clinical Trials of Acupuncture) [49].

#### PLWNT Group

**Figure 2:**
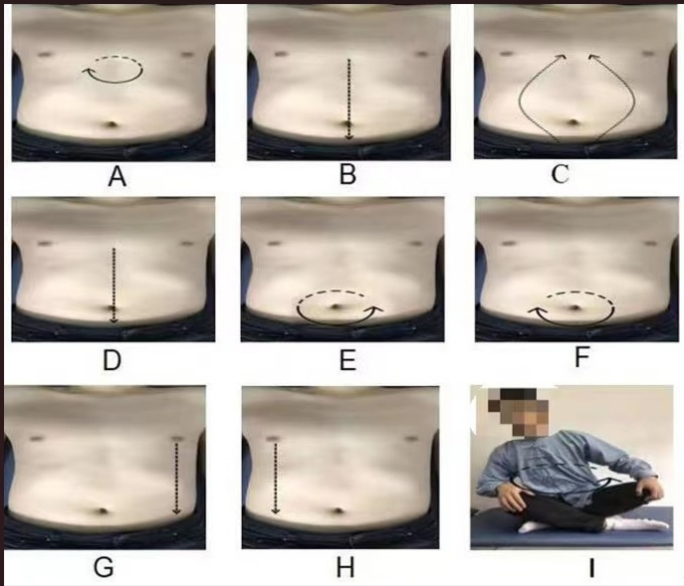
(A) Press and knead acupoint in Danzhong. (B) Rubbing from Danzhong Acupoint to Pubic Symphysis. (C) Rubbing from Pubic Symphysis to Danzhong Acupoint. (D) Pushing from Danzhong Acupoint to Pubic Symphysis. (E) The right-hand massages the abdomen by the left circle. (F) The left-hand massages the abdomen by the right circle. (G) Pushing with the right hand from the left breast to the groin. (H) Pushing with the left hand from the right breast to the groin. (I) Turn left and right. Every movement will be carried out 21 times. PLWNT: prolong life with nine turn.

The PLWNT intervention program and operating standards refer to the Chinese general higher TCM compiled from the college textbooks of Tuina and Qigong. Experienced Qigong teachers at Shanghai University of TCM, who have been teaching Qigong for at least 5 years, will be placed in charge of the supervision of the exercise, and they will correct participants’ exercise postures during the entire intervention period for 1 h every Sunday, including the first 10 min of each session for stretching and relaxation exercises, as well as movement introductions and demonstrations. The subsequent 20 min are allotted for individual guidance and correction of actions. Finally, all participants will practice PLWNT for 30 minutes together. For the remaining 6 days of the week, all participants will practice by themselves for 30 minutes at 6 PM o’clock every day at home, under the remote supervision of one of the directors. The practice videos will be required to be posted in a WeChat group comprising all of the study participants. If some participants find this inconvenient, videos can be sent privately to the study investigators. All participants will be asked to write down their feelings in a practice recording notebook after each exercise. The average amount of abdominal stimulation for the first 8 rubbings was 0.5 (SD 0.1) kg, and the strength of the stimulation will be monitored in real time in the LABVIEW2017 software (SHUTCM) so that the patients can feel the amount of stimulation. The entire practice process will last for 12 weeks. The content of the PLWNT Qigong intervention is the same as that reported in our previous research The 9 specific forms of manipulation are shown in Figure 2 [39,50].

#### CBT Group

Qualified CBT therapists with professionally accredited qualifications will be invited to conduct CBT for 1 h each week by giving lectures or psychological consultations on the prevention and treatment of CFS. The sessions will cover CFS diagnosis, diet and nutrition, life management, health and lifestyle changes, physical and mental health education, and medical management. On the remaining 6 days of the week, all participants will be required to listen to lectures on WeChat for 30 minutes every day. If some participants find this inconvenient, they will be allowed to learn at their own pace using the PowerPoint (Microsoft Corp) presentations provided. Each participant will be asked to write down their feelings in a practice recording notebook after each web-based session to ensure that the other conditions are the same as those of the PLWNT group. The entire practice process will last for 12 weeks. Detailed information is available in a previously published paper [39,50]; however, in this research, we will focus on CBT treatment in the gastrointestinal and nervous system fields.

### Outcome Measures

The primary outcome is the MFI-20, while secondary outcomes will include the Pittsburgh Sleep Quality Index (PSQI), Gastrointestinal Symptom Rating Scale (GSRS), Hospital Anxiety and Depression Scale (HADS), fMRI, gut microbiota, and peripheral blood. The specific study outcome assessment time points are shown in Table 1.

**Table 1:** Time points of the outcomes assessment.

			Screening (T-1)^a^		Intervention								Follow up (T20)
					T0		T4	T8		T12			
Inclusion criteria			✓										
Exclusion criteria			✓										
Informed consent			✓										
Randomization					✓								
**Outcomes**													
	MFI-20^b^	✓		✓		✓		✓	✓		✓		
	SF-36^c^	✓		✓		✓		✓	✓		✓		
	PSQI^d^	✓		✓		✓		✓	✓		✓		
	GSRS^e^	✓		✓		✓		✓	✓		✓		
	HADS^f^	✓		✓		✓		✓	✓		✓		
	fMRI^g^			✓					✓				
	gut microbiota			✓					✓				
	peripheral blood			✓					✓				

^a^T0-T20: Time.

^b^MFI-20: Multidimensional Fatigue Inventory 20.

^c^SF-36: Short Form 36-item Health Survey.

^d^PSQI: Pittsburgh Sleep Quality Index.

^e^GSRS: Gastrointestinal Symptom Rating Scale.

^f^HADS: Hospital Anxiety and Depression Scale.

^g^fMRI: functional magnetic resonance imaging.

#### Primary Outcome

The MFI-20 is widely used for the measurement of mental and physical fatigue in patients with CFS [51]. It comprises 20 items, with 5 dimensions, that is, general fatigue, physical fatigue, mental fatigue, reduced activity, and reduced motivation. The total possible score is 100 points, where the higher the score, the more severe the fatigue. These will be measured at weeks 0, 4, 8, and 12.

#### Secondary Outcome

##### HADS

The HADS is used to evaluate the degree of anxiety and depression in patients. The scale consists of 14 items, including 7 items that assess anxiety and 7 items that assess depression [52]. The higher the total score, the more severe the degrees of anxiety and depression.

##### PSQI

The PSQI is a self-assessment questionnaire used to evaluate sleep quality. The scale consists of 24 items, including subjective sleep quality, sleep latency, sleep duration, habitual sleep efficiency, sleep disturbances, use of sleeping medication, and daytime dysfunction [53]. The score range for each dimension is from 0 to 3, with a total possible score of 21, where the higher the score, the poorer the sleep quality.

##### GSRS

The GSRS is mainly used to assess the severity of gastrointestinal symptoms within the past week. The GSRS includes 15 common gastrointestinal symptoms, which are divided into 5 symptom groups: reflux, abdominal pain, indigestion, diarrhea, and constipation. In addition to assessing the pain, soreness, and indigestion of the upper gastrointestinal tract, stool characteristics and discomfort of the lower gastrointestinal tract can be evaluated. Each question is scored from 1 to 7, where the higher the score, the more severe the symptoms.

##### fMRI

**Figure 3:**
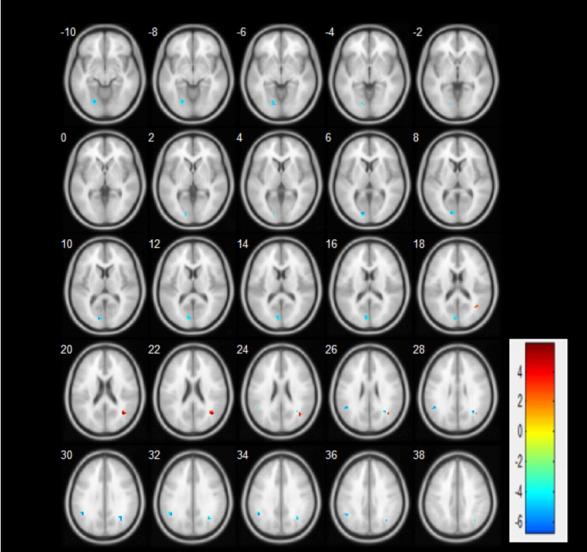
Based on the results of the previous data, we speculated that the brain regions that may be activated include the temporal lobe, the frontal lobe, and the parietal lobe.

fMRI data will be obtained from all participants using the 3.0-T Trio Siemens System at the Shanghai Municipal Hospital of Traditional Chinese Medicine. Thirty-two head coils will be used for scanning. In the resting state BOLD signal acquisition single excitation gradient echo-plane imaging sequence, the participants will be scanned using the following parameters: repetition time (*T*_R_)=1900 milliseconds; effective echo time (*T*_E_)=2.93 milliseconds; sagittal slices=188; thickness/skip=1.2/0.6 mm; field of view (FOV)=256×256 mm^2^; matrix=240×256 mm^2^; voxel size=1.0×1.0×1.0; phase encoding direction=A>>P; and flip angle (*F*_A_)=90°. The participants will be asked to close their eyes, rest for 10 min, and not think about anything before the scan. The participants will be instructed to not move their heads during data collection. The 2 groups of participants will be tested before and after the experiment. Based on the results of previous studies, we speculated that the brain regions that may be activated include the temporal lobe, frontal lobe, and parietal lobe, which are shown in Figure 3.

##### Gut Microbiota

For stool specimen collection, we will collect approximately 5 g of fecal specimen per research participant within 1 week of enrollment. The sample will be placed in a 40-mL sterile fecal drying box, immediately sealed, and numbered, before transporting it in a heat preservation box with an ice pack within 1 h of collection and storing it in a refrigerator at –80°C. The samples will be sent for inspection after all of the specimens have been collected. Approximately 200-300 mg of stool specimens will be extracted using the stool FastDNA kit (MP Bio), and the 16S rRNA gene will be amplified with specific primers (16S V4: 515F-806R). All polymerase chain reactions will be performed using Phusio high-fidelity PCR Master Mix (New England Biolabs), and the library will be sequenced on the Illumina HiSeq 2500 platform. The 16S rRNA sequencing data will be analyzed using the Quantitative Insights into Microbial Ecology platform quantitative analysis (version 1.9.1; Majorbio Inc), and we will select operating taxonomic units with a similarity threshold of 97%. We will use the serial number standard corresponding to the sample with the least sequence to normalize the abundant information of operating taxonomic units. For each representative sequence, the classification information is marked according to the GreenGene database of the RDP classifier algorithm.

### Statistical Analysis

SPSS (version 25.0; SPSS Inc Chicago) will be used for statistical analysis. The baseline characteristics of patients in each group will be analyzed descriptively. All numerical data will be expressed as the average value, X̄ (SD). Quantitative data that do not fit a normal distribution will be expressed as the median (IQR). In all analyses, statistical significance will be set as a 2-tailed *P*<.05. For the primary outcome, CFS scores will be assessed using a linear mixed-effects model with time and group interaction effects. Bonferroni correction will be used to account for multiple comparisons.

For fMRI analysis, we will select the largest peak coordinate of the brain area with a difference after amplitude of low frequency fluctuations analysis, save the block where the peak coordinate is located as the mask as the region of interest, and count the difference in the functional connection density between the region of interest and the whole brain. The specific process is to make a functional connection between the seed area (amplitude of low frequency fluctuation difference brain area) and all voxels of the whole brain. Each voxel will be given a correlation value, and the normal distribution *z* scores of the 2 sets of images will be obtained using Fisher transformation, (where r is the correlation coefficient):

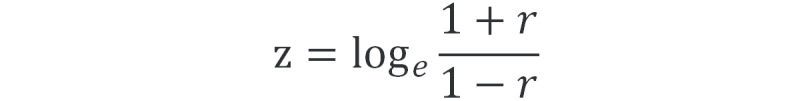



That is, the functional connectivity density average image. Two-sample 2-tailed *t* test will be performed between the groups, and a paired *t* test was performed within the groups. *P* values less than .05 will be taken to indicate statistical significance and the brain regions will be corrected by FWE, while XJVIEW will be used to present the results.

### Adverse Events

All participation in this study will be entirely voluntary, and all participants will be required to sign a written informed consent, including their rights and possible benefits and risks. Participants can refuse to participate in the study or withdraw from the study at any time during the study. If participants choose to withdraw from this study, they will not be affected, and they will not be discriminated or retaliated against.

Adverse events (AEs) can be any adverse and insignificant experimental results, symptoms, or research-related diseases [54]. Most studies have shown that Qigong treatment is a relatively safe method, with no serious AEs reported [55-57]. If AEs occur, they are defined as any damage caused by the intervention, such as headache, dizziness, chest tightness, heart palpitations, muscle aches, neurasthenia, and psychological stress. Regardless of whether it is related to treatment, the affiliated doctor of the Shanghai Municipal Hospital of Traditional Chinese Medicine will be notified on time and the AE will be recorded on the case report form. The doctor will make a corresponding judgment and start treatment. If serious AEs occur, the investigator will report those to the main investigator and the ethics committee to determine whether the participant should be withdrawn from the study and treatment. The study will be suspended for further instructions if necessary. The researchers will do their best to prevent and deal with possible damages caused by this research. If the expert committee believes that AEs are related to Qigong treatment, the research team will provide treatment costs and corresponding financial compensation for damages related to the trial.

### Confidentiality

To protect confidentiality, all participants will be classified by unique codes instead of names. The identity information of all participants will be kept confidential such that only the researchers will be allowed access to it. Following completion of the study, the participants’ data will be securely locked for 2 years and then destroyed under the protection of the management department. Copies of the participants’ feces and blood will be stored for 2 years with the participants’ consent.

### Quality Control

Qigong intervention plans and operating standards refer to the relevant content of the nationally compiled textbook for higher education in traditional Chinese medicine and make standard videos. Quality control will be conducted under the supervision of an independent steering committee, comprising investigators with experience in Qigong clinical trials, statistical experts, and experts in exercise and AEs, to eliminate potential bias and ensure the quality of the trial. The steering committee will be responsible for monitoring the data, identifying problems, reviewing the data collected, and controlling for bias. All instructors and evaluators must be certified to have been teaching Qigong or psychology for at least 5 years and must undergo a standardized training program conducted by a nationally recognized academic expert with at least 10 years of experience in Qigong training before the start of the trial. The training program shall be conducted in strict accordance with the PLWNT protocol, including PLWNT forms, principles, breathing techniques, concentration, and CFS precautions. Two groups will be conducted simultaneously, with a minimum of 10 people in each group, to avoid variations in the severity of CFS due to the season and weather. Participants must upload photos or videos of their home practice the remaining 6 days of the week to provide feedback to the instructor and will be encouraged via the WeChat app to complete their daily Qigong practice. The participants will not be assigned additional exercises during the study to maintain consistency in individual PLWNT strengths. At the time of enrollment, patients will undergo a 3-day training and stimulation calibration by wearing a morcellation stimulation glove. The average amount of abdominal stimulation for the first 8 rubbings will be 0.5 (SD 0.1) kg, and the strength of the stimulation will be monitored in real time using LABVIEW2017 software, so that the patients can feel the amount of stimulation. If necessary, the Qigong instructor will adjust the number and range of PLWNT movements to achieve the intended intensity.

### Follow-Up

After the study, the participants will resume their original life in unsupervised mode, but they will need to record daily exercise or physical activity information and send this information to the researchers by email every day. The researchers will gather the participants once a week to discuss CFS-related issues and detailed follow-up actions. They will also give gifts such as traditional Chinese medicine sachets, exercise clothing, and pain patches to thank the participants and increase their enthusiasm for follow-up. All indicators will be reassessed at 8 weeks after the end of the trial to determine the long-term efficacy.

### Ethical Considerations

This study will be conducted in accordance with the Declaration of Helsinki and the International Code of Ethics for Biomedical Research Involving Human Subjects. The study was approved in March 2022 by the Ethics Committee of Shanghai Municipal Hospital of Traditional Chinese Medicine (Ethics Approval Number 2022SHL-KY-05) and registered in the China Clinical Trials Registry (ChiCTR2200061229). Informed consent will be obtained before enrollment. Before consent seeking, we will introduce the study and explain its purpose. The study data will be anonymized. At the end of the study, A set of Tai Chi clothing for practicing Qigong is provided for free.

## Results

The study was approved in March 2022 by the ethics committee of Shanghai Municipal Hospital of Traditional Chinese Medicine (Ethics Approval Number 2022SHL-KY-05). Recruitment started in July 2022. The intervention is scheduled to be completed in December 2024. Data collection will be completed by the end of January 2025. Over the 3-year recruitment period, 208 participants will be recruited. Data management is still in progress; therefore, data analysis has yet to be performed.

## Discussion

CFS is a complex multisystem disease with unknown etiology. Compared with healthy individuals, individuals with CFS exhibit altered emotion regulation, somatic and sensory processing, and motor control activities [58]. Recommended drug therapy can only partially relieve fatigue and may aggravate other symptoms. Qigong therapy is a mild, low-intensity, mind-body aerobic exercise that emphasizes a combination of physical posture, breathing concentration, and mental concentration, which is a relatively safe and more effective treatment for CFS than other treatments [59]. In recent years, increasing research has been conducted on Qigong for the improvement of the digestive system, endocrine system, and so on [60,61]. Therefore, the aim of this study is to evaluate the efficacy of PLWNT on fatigue, insomnia, anxiety, and gastrointestinal symptoms in patients with CFS and fully explore the mechanisms of brain-gut regulation in the context of CFS.

PLWNT includes several physical and mental exercise methods. The National Institutes of Health considers it a mobile meditation. The abdominal technique included in PLWNT involves the use of meridians and collaterals to regulate organs [62]. The whole process directly massages and stretches the internal organs to stimulate the nerve receptors on the gastrointestinal and mesentery. The PLWNT also focuses on the influence of the direction of the abdomen on the inherent movement of the large and small intestines, stimulates the nerve conduction pathway and the nerve-emotion pathway, and strengthens the connection between the digestive system and the cranial nerves to improve the sleep and gastrointestinal symptoms of patients with CFS, with the help of the heat of friction between the palms, it warms the spleen and yang, assists the righteous qi, promotes the circulation of qi and blood in the whole body, strengthens the interaction between the brain and the intestines, enhances the static strength of muscles, coordinates the autonomic nervous system, and increases blood circulation to the heart, which alleviates fatigue and sleep disturbance and improves the quality of life of patients with CFS [63,64]. In our previous study, PLWNT was found to alleviate fatigue and improve the quality of life of patients with CFS [39]. However, to date, there has been no study on the regulation of CFS fatigue, insomnia, anxiety, and gastrointestinal symptoms by PLWNT Qigong from the perspective of brain-gut regulation.

Regarding the brain-gut regulation mechanism of PLWNT intervention in CFS, various hypotheses have been proposed to explain the interaction between the gut and brain, but special attention has been paid to imaging of neuropeptides, inflammatory mediators, intestinal microbiota, and brain function [65,66]. Increasing evidence shows that the diversity of intestinal flora is essential not only for the health of the intestine but also for the general physiological functions of other organs, especially the brain [67,68]. A recent study found differences in the composition and function of the gut microbiota between patients with CFS and healthy controls [69,70]. In addition, previous fMRI studies have confirmed that the brain network structure and connectivity of patients with CFS are abnormal [71,72] and the changes in the composition profile of the intestinal flora are related to changes in the cingulate and cerebellum signals involved in brain activity [73]. Several studies related to the influence of Qigong exercise on the structural characteristics of intestinal flora have shown that Qigong can substantially increase the diversity of intestinal flora and the content of beneficial bacteria [74,75]. Qigong can also relieve sleep, anxiety, and depression symptoms, while its beneficial effect on gastrointestinal function may be related to brain function connection and central nervous brain area response [76]. Combining neuroimaging and intestinal microbiology analysis, exploring the brain-gut regulation mechanism of CFS treatment represents a novel research direction In this study, we will use fMRI to analyze the functional connectivity density of patients with CFS and its relationship with intestinal flora to explore changes in brain and bowel activity. In addition, the MFI-20, PSQI, HADS, and GSRS will be used to explore the changes in fatigue, sleep, mental, and gastrointestinal symptoms before and after PLWNT intervention. The benefits of PLWNT have been reported previously; however, most studies only assessed self-reported symptoms by patients. Therefore, in this study, we aimed to explore the efficacy and safety of PLWNT for treating CFS from the objective perspective of brain-gut regulation, with the aim of providing high-quality treatment options and optimized guidance for patients with CFS.

First, ideally, patients should be unaware of their group assignment, but this is difficult to achieve in nondrug trials, so psychological effects may inevitably occur. However, we will endeavor to ensure that laboratory technicians, data managers, and statisticians are aware of patient recruitment and processing. Second, this study will include patients between the ages of 20 and 60 years; therefore, the results cannot be generalized to adults older than 60 years. Future studies will use interventions that control for all nonspecific factors to better understand the specific efficacy of PLWNT.

PLWNT is easy to learn and self-manage and is not limited by time and space. If PLWNT can improve the clinical symptoms of insomnia, anxiety, and gastrointestinal discomfort in patients with CFS, it should be promoted in the community in the future. The findings of this study will help determine whether PLWNT has a better effect than CBT for treating patients with CFS and will provide more secure, more effective CFS treatments.

## Abbreviations

AE: adverse event
CBT: cognitive behavioral therapy
CFS: Chronic fatigue syndrome
CONSORT: Consolidated Standards of Reporting Trials
FA: flip angle
fMRI: functional magnetic resonance imaging
FOV: field of view
GSRS: Gastrointestinal Symptom Rating Scale
HADS: Hospital Anxiety and Depression Scale
MFI-20: Multidimensional Fatigue Inventory 20
PLWNT: prolong life with nine turn
PSQI: Pittsburgh Sleep Quality Index
SPIRIT: Standard Protocol Items: Recommendations for Intervention Trials
STRICTA: Standards for Reporting Interventions in Clinical Trials of Acupuncture
TE: echo time
TR: repetition time

## Data Availability

Data sharing is not applicable to this article as no data sets were generated or analyzed during this study.
